# The role of cognitive flexibility in moderating the effect of school-related stress exposure

**DOI:** 10.1038/s41598-023-31743-0

**Published:** 2023-03-31

**Authors:** Orly Harel, Alla Hemi, Einat Levy-Gigi

**Affiliations:** 1 Faculty of Education, Bar Ilan University, Ramat Gan, Israel; 2 The Gonda Multidisciplinary Brain Research Center, Bar Ilan University, Ramat Gan, Israel; 3 Faculty of Education and the Brain Science Center, Bar Ilan University, Ramat Gan, Israel

**Keywords:** Environmental social sciences, Psychology and behaviour

## Abstract

Educators are exposed to various stressful events as part of their ongoing work, including violence, sexual assaults, suicidal behavior, and loss or illness of students or their family members. Previous studies revealed an increased risk of developing PTSD symptoms in healthcare and supportive professionals exposed to repeated stress as part of their work. Cognitive flexibility might be a protective factor against the negative effect of such stress. The current study aimed to examine the interactive effects of school-related stress exposure and cognitive flexibility on the tendency to develop Post Traumatic Stress Disorder (PTSD) symptoms and the coping abilities of educators. One hundred and fifty educators (86.5% female; *M*_age_ = 43.08, *M*_teaching_experience_ *=* 12.90) volunteered to participate in this study. They completed questionnaires measuring levels of stress exposure, cognitive flexibility, coping ability, and PTSD symptoms. Analyses revealed that cognitive flexibility moderated the relationship between school-related stress exposure and both PTSD symptoms severity and maladaptive coping. Specifically, whereas educators with low cognitive flexibility exhibited positive associations between continuous stress exposure and both levels of PTSD symptoms and maladaptive coping, no such association was found among educators with high cognitive flexibility. The results highlight the importance of cognitive flexibility as a protective factor against the harmful effects of possible stress exposure in school environments. Awareness of the crucial role of cognitive flexibility as a protective factor for educators can be a breakthrough in improving educators' well-being and adaptive functioning.

## Introduction

Many studies have examined Post Traumatic Stress Disorder (PTSD) symptoms in individuals exposed to continuous stress as part of their daily professional routine, including studies on first responders, social workers, therapists, and other professionals in the help and care service^1–6^. Whereas educators are continuously exposed to potentially stressful events as part of their ongoing work at school, only a few studies have examined their tendency to develop PTSD symptoms^7,8^. Here we test, for the first time, how levels of stress exposure interact with cognitive flexibility skills to predict the tendency to develop PTSD symptoms and the ability to cope with aversive events adaptively.

Previous studies have shown that both direct and indirect stress exposure is associated with enhanced PTSD symptoms (for review, see^9^). Indeed, educators are directly exposed to various crises and stressful situations (e.g. verbally or physically violent incidents between students or against the personnel, suicidal attempts) as well as to indirect stress while providing social-emotional support to students and their families who are experiencing stressful and traumatic incidents (e.g. death or illness in the family, immigration-related trauma)^10–17^.

It is common knowledge that exposure to stressful and traumatic incidents may lead to the development of PTSD symptoms. However, the relationship between the level of exposure and the tendency to develop symptoms is not yet clear. Several studies in trauma-exposed individuals revealed a positive relationship between exposure and clinical symptoms^1–3,18–21^. These studies suggest a cumulative negative effect of traumatic exposure, in which more exposure results in increased PTSD severity. Other studies report seemingly contradicting results, pointing to a negative association between exposure and clinical symptoms^22–25^. Accordingly, it is possible that, at some point, individuals habituate to stress in a way that eliminates its possible deleterious effects. These mixed results may suggest that the relationship between the level of exposure to stressful and traumatic exposure and the tendency to develop PTSD symptoms is more complex. Indeed, some studies on educators, as well as on other professionals exposed to repeated stressful situations, such as firefighters, police officers, and nurses, show that while they may develop PTSD symptoms^7,8,12–14,16,26–28^, there are individual differences in this tendency^4,29^.

Previous studies conducted in our lab suggest that cognitive flexibility, defined as the ability to update prior beliefs, cognitions, and behaviors according to contextual demands^30,31^, may play a moderative role in the relationship between repeated traumatic exposure and the tendency to develop clinical symptoms including PTSD and depression among young adults who served in the army^32,33^. It was found that individuals with lower levels of cognitive flexibility exhibit a positive association between exposure and symptoms. Hence, greater traumatic exposure is associated with higher clinical symptoms. On the other hand, individuals with higher levels of cognitive flexibility show no such association. Similarly, two different studies on trauma-exposed adults showed that cognitive flexibility is a significant protective factor that reduces the severity of PTSD symptoms and improves psychological well-being following traumatic incidents^34,35^. Hence, we expect cognitive flexibility to play a similar role in the relationship between school-related stress and PTSD symptoms in educators.

One additional factor that plays a significant role in how educators function under stress is their ability to cope with the internal and external demands of potentially stressful situations^36–39^. Studies in this population revealed that employing adaptive coping strategies was associated with a better ability to function in school. In contrast, maladaptive coping increased the risk for psychological distress, including depression, anxiety, and somatic symptoms, as well as elevated alcohol consumption, burnout, and turnover intentions^39–47^. While these studies reveal a robust association between coping and functioning, according to our knowledge, no study has tested the interactive effect of stress exposure and cognitive flexibility on coping abilities. Traditionally, it is common to distinguish between two aspects of coping^48^. One emphasizes coping as a personality characteristic. The other refers to coping as a tool to manage stress over time and in changing situations. This aspect implies a theoretical link between coping and flexibility since both require choosing one adequate strategy out of various options that meet situational demands.

Previous studies in young adults revealed that high levels of cognitive flexibility are associated with choosing adaptive coping strategies. In contrast, low levels of cognitive flexibility are associated with choosing maladaptive coping strategies^49–51^. Moreover, coping abilities are positively correlated with coping skills in healthcare providers^52^. Hence, the current study aims to test the role of cognitive flexibility in the relationship between school-related stress exposure and the tendency to develop PTSD symptoms, as well as the ability to cope with given situations adaptively. In line with the existing literature, we hypothesized that cognitive flexibility would moderate these relationships, showing that high levels of cognitive flexibility serve as protective factors against the cumulative effect of stress.

## Method

### Participants and procedure

The sample size was calculated using G*Power software^53^. Based on the effect size found in a previous related study^54^, we conducted an a-priori power analysis for the moderation models. This revealed a need for 137 participants based on the ability to detect a medium-size effect (Cohen’s *f* = 0.25) in the study, with a 5% significance level (α) and 85% power level (1-β)^55^. We increased the sample size by 20% to account for possible participant dropout or technical problems. Based on these results, we recruited 168 participants. Seven participants (4.2%) did not complete the coping scales, 5 (3.0%) did not complete the PCL scale, and 6 participants (3.6%) encountered technical problems completing the questionnaires. This sums up to 18 participants who were not included in the analyses. Importantly, these participants did not differ from the other participants in gender, age, or levels of work-related stress exposure. The remaining 150 educators (86.5% female; *M*_age_ = 43.08, *SD* = 9.83) had no missing data and were included in the analyses. The average number of years as an educator in the sample was 12.90 (*SD* = 11.24). 73% were married, 15% were single, 9% were divorced, and 1% were widowed, and 2% of the participants defined their marital status as “other.” Participants were recruited by contacting elementary and secondary school principals in the center of Israel and receiving their approval to recruit homeroom teachers and educational counselors from their schools. The experimenter arrived at the participating schools and provided information about the aims of the study and its procedure. All participants provided written informed consent. Then they received a link to an online survey powered by Qualtrics. The study was approved by the ethics committee of the Faculty of Education of Bar Ilan University (approval number 69). All research was performed following the guidelines of the ethics committee and by the Declaration of Helsinki.

### Measures

We used the following self-report questionnaires to assess continuous levels of work-related stress exposure, cognitive flexibility, coping, and PTSD symptoms (see Table 1 for reliabilities of the scales). For all questionnaires, we used a previously validated Hebrew version (e.g.^56^).

**Table 1:** Zero-order correlations of the study variables.

Variable	School-related stress exposure	Cognitive flexibility	PTSD symptoms	Adaptive coping
School-related stress exposure				
Cognitive flexibility	0.02			
PTSD symptoms	0.21^**^	− 0.12		
Adaptive coping	0.06	0.35^***^	− 0.11	
Maladaptive coping	0.06	− 0.15	0.29^***^	0.06

^*^p < 0.05, ^**^p < 0.01, ^***^p < 0.001.

#### Work-related stress exposure

To assess the level of accumulative stress exposure in a given month at school, we adopted a well-validated scale^57,58^, and revised it to match the population of interest (for a similar approach, see^27^). To that end, we first interviewed 12 educators and chose the ten most common stress-related incidents (e.g. “A parent being physically or verbally violent towards the staff”). In the questionnaire, participants had to rate the frequency of their exposure to each of these ten incidents on a 1 (not exposed) to 6 (exposed at least a few times in a representative week) Likert scale (for studies using this measure in other populations see^59–61^). The reliability of the scale was α = 0.82.

#### Cognitive flexibility scale (CFS)

Cognitive flexibility was assessed by a self-report questionnaire that comprised 12 items (e.g. “I avoid new and unusual situations”) on a scale from 1 (“Strongly disagree”) to 6 (“Strongly agree”)^62^. The CFS scale is a widely used self-report measure of cognitive flexibility (e.g.^54,63,64^). However, it should be noted that, like other self-report measures, it does not always correlate with the neuropsychological assessment of cognitive flexibility (for further discussion, see^65^). The reliability of the scale was α = 0.82.

#### Coping ability (brief COPE)

Coping was measured using eight items from the Brief COPE questionnaire^66^. We assessed two scales, adaptive coping (e.g. “I’ve been concentrating my efforts on doing something about the situation I’m in”) and maladaptive coping (e.g. “I’ve been giving up the attempt to cope”). Following Meyer^67^, we focused primarily on maladaptive coping as we aimed to examine the protective role of cognitive flexibility against maladaptive outcomes. The participants were asked to rate their level of agreement with each item on a Likert scale of 1 (“Do not agree”) to 4 (“Totally agree”). The reliability of the scale was α = 0.62.

#### PTSD symptoms (PCL-5)

PTSD symptoms were assessed using a 20-item self-report checklist based on the DSM-5 criteria addressing symptoms during the past month (e.g. “In the past month, how much were you bothered by recurring, troubling, and unwanted memories of the stressful experience?”)^68,69^. Participants were asked to choose a response on a Likert scale of 0 (“Not at all”) to 4 (“Extremely”). The reliability of the scale was α = 0.95.

### Statistical analyses

Data were analyzed using the statistical software SPSS version 27 (Chicago, IL). Descriptive statistics and correlations were calculated between all variables in the present study (see Table 1). An α = 0.05 was used for all statistical analyses. Moderation analyses were conducted using Hayes’s^70^ PROCESS macro. In both moderation models, school-related stress exposure and cognitive flexibility were entered as independent and moderator variables, respectively. Age and gender were entered as control variables. Only participants with values on all scales included in the models were included in the analyses (listwise deletion).

## Results

Zero-order correlations were conducted between all the study variables indicating that school-related stress exposure was positively correlated with PTSD symptoms (Table 1). In addition, cognitive flexibility was positively correlated with adaptive coping. Finally, PTSD symptoms were negatively correlated with maladaptive coping.

### School-related stress exposure, cognitive flexibility, and PTSD

**Figure 1:**
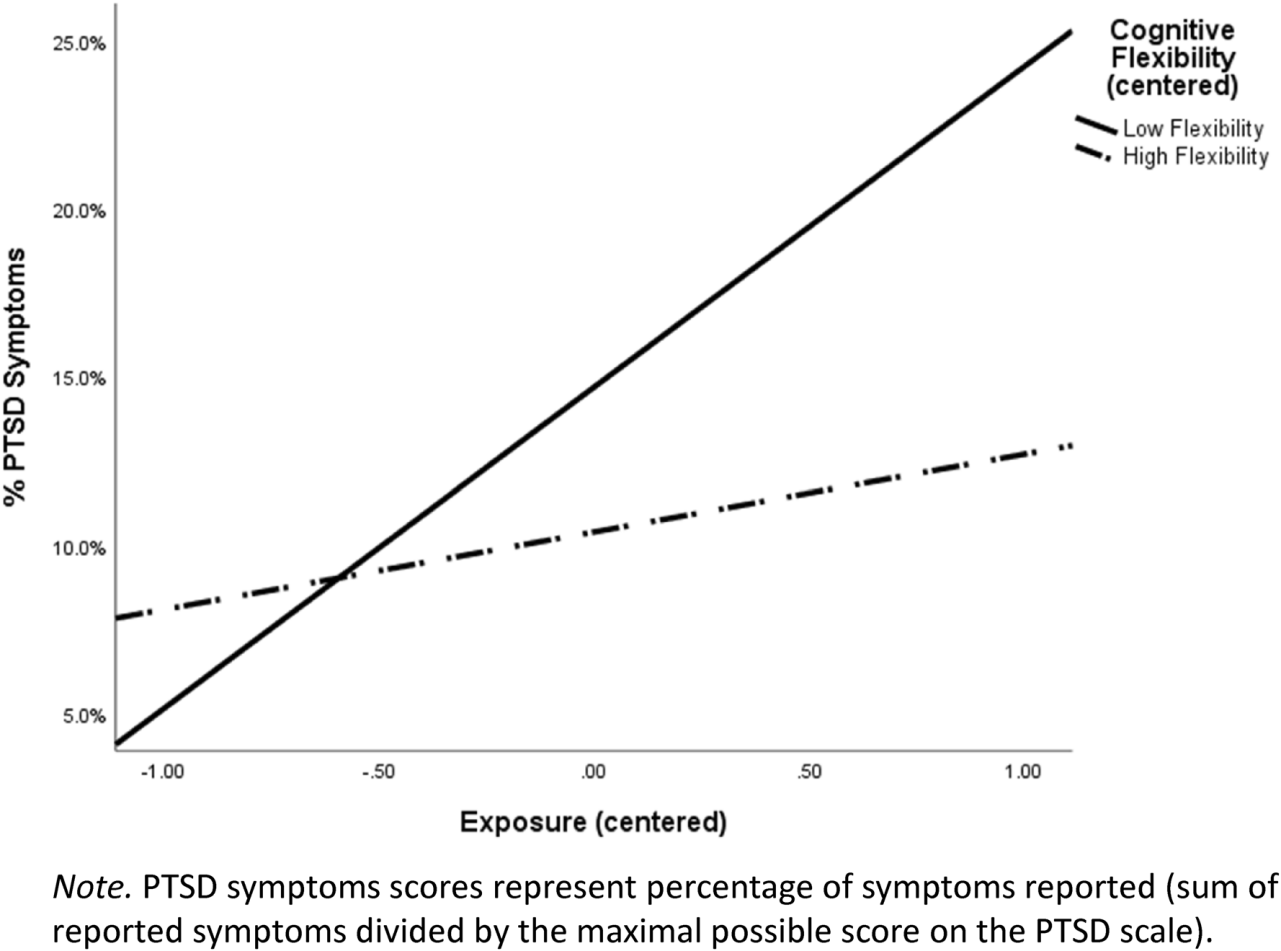
The interactive effect of school-related stress exposure and cognitive flexibility on PTSD symptoms in educators.

To test the hypothesis that cognitive flexibility moderates the relationship between school-related stress exposure and PTSD symptoms, we employed Hayes’s PROCESS macro^70^. In this model (Table 2), school-related stress exposure, cognitive flexibility, and PTSD symptoms were entered as independent, moderator, and outcome variables. Age and gender were entered as control variables. The general model was significant (*R*^2^ = 0.13, *F* (5, 143) = 4.42, *p* < 0.001). Core analyses revealed a significant main effect of school-related stress exposure. Specifically, higher levels of stress exposure were associated with higher PTSD symptoms. Additionally, consistent with our hypothesis, there was a significant interactive effect of school-related stress exposure and cognitive flexibility on the severity of PTSD symptoms. This interaction accounted for an additional 3.94% of the variance beyond the variance explained by the main effect. To interpret this interactive effect, we computed bootstrapping confidence intervals (95%), evaluating the magnitude of the relationship between stress exposure and PTSD symptoms severity for participants with low and high levels of cognitive flexibility. As predicted, for participants with low levels of cognitive flexibility (SD = − 1), the results revealed a significant positive relationship between stress exposure and severity of PTSD symptoms (*B* = 0.38, CI 95% [0.20, 0.56], *t* (149) = 4.12, *p* < 0.001). However, for participants with high levels of cognitive flexibility (SD = 1), no such connection was observed (*B* = 0.11, CI 95% [− 0.02, 0.23], *t* (149) = 1.73, *p* = 0.09) (See Fig. 1).

**Table 2:** The interactive effect of school-related stress exposure and cognitive flexibility on PTSD symptoms severity (estimated coefficients, standard errors, and 95% confidence intervals).

Variables	*B*	S.E.	*t* value	95% CI	
				Low	High
Control variables					
Gender	− 0.01	0.15	− 0.07	− 0.31	0.29
Age	0.00	0.01	0.16	− 0.01	0.01
Predicting variables					
School-relates stress exposure	0.24	0.06	4.25^***^	0.13	0.36
Cognitive flexibility	− 0.12	0.07	− 1.73	− 0.27	0.02
Stress exposure * cognitive flexibility	− 0.20	0.08	− 2.55^*^	− 0.36	− 0.05

*CI* confidence interval.

^**^p < 0.01, ^***^p < 0.001.

### School-related stress exposure, cognitive flexibility, and coping

**Figure 2:**
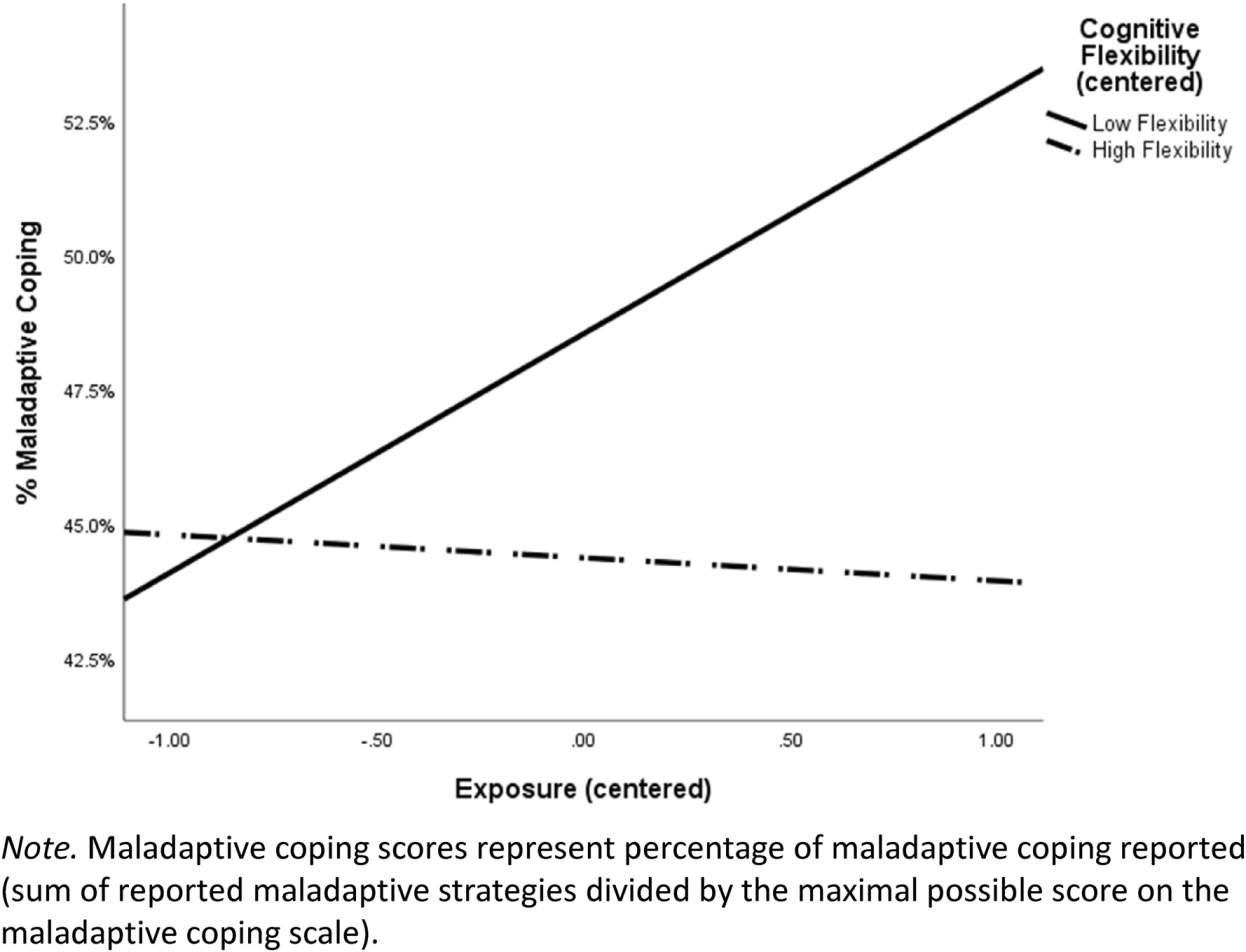
The interactive effect of school-related stress exposure and cognitive flexibility on maladaptive coping.

To test the hypothesis that cognitive flexibility moderates the relationship between school-related stress exposure and coping, we employed Hayes’s PROCESS macro^70^. In this model (Table 3), school-related stress exposure, cognitive flexibility, and maladaptive coping (An additional model was assessed for adaptive coping as a dependent variable. No significant interactive effect emerged for the combined effect of school-related stress exposure and cognitive flexibility on adaptive coping.) were entered as independent, moderator, and outcome variables, respectively. Age and gender were entered as control variables. The general model was significant (*R*^2^ = 0.09, *F* (5, 143) = 2.82, *p* = 0.02). Core analyses revealed a significant main effect of stress exposure, indicating higher levels of stress exposure were associated with higher levels of maladaptive coping. Importantly, consistent with our hypothesis, stress exposure and cognitive flexibility had a significant interactive effect on the level of maladaptive coping. This interaction accounted for an additional 3.52% of the variance beyond the main effects’ explanation. To interpret the interactive effect, we computed bootstrapping confidence intervals (95%), evaluating the magnitude of the relationship between stress exposure and level of maladaptive coping ability for participants with low and high levels of cognitive flexibility. As predicted, for participants with low levels of cognitive flexibility (SD = − 1) there was a significant positive relationship between stress exposure and level of maladaptive coping (*B* = 0.78, CI 95% [0.20, 1.36], *t* (149) = 2.66, *p* = 0.009). On the other hand, for participants with high levels of cognitive flexibility (SD = 1), no such correlation was found (*B* = − 0.02, CI 95% [− 0.41, 0.36], *t* (149) = − 0.12, *p* = 0.91) (See Fig. 2).

**Table 3:** The interactive effect of school-related stress exposure and cognitive flexibility on maladaptive coping (estimated coefficients, standard errors, and 95% confidence intervals).

Variables	*B*	S.E.	*t* value	95% CI	
				Low	High
Control variables					
Gender	0.19	0.48	0.38	− 0.77	1.14
Age	− 0.03	0.02	− 1.86	− 0.07	0.00
Predicting variables					
School-relates stress exposure	0.38	0.18	2.07^*^	0.02	0.74
Cognitive flexibility	− 0.43	0.23	− 1.87	− 0.88	0.02
Stress exposure * cognitive flexibility	− 0.59	0.25	− 2.35^*^	− 1.09	− 0.09

*CI* confidence interval.

^*^p < 0.05.

## Discussion

The present study aimed to examine the interactive effect of school-related stress exposure and cognitive flexibility on the tendency to develop PTSD symptoms and on the coping abilities of school educators. Studies over the years have dealt with the stress of educators. Their stress has been found to be at higher-than-average levels and has been found to have physical and emotional effects not only on the educators themselves^42,71^ but also on the social and emotional functioning of the students^26^. However, it was unclear whether such school-related stress has similar implications as it has in other professions (e.g. healthcare personnel and first responders).

The results of the current study revealed a significant positive correlation between school-related stress exposure and PTSD symptom severity. This finding is consistent with previous studies, which showed a similar relationship between stress and PTSD in social workers^2,22^ and nurses^72^. Additionally, in line with our hypothesis, we found a significant interactive effect of school-related stress exposure and cognitive flexibility on the severity of PTSD symptoms. Specifically, for educators with low levels of cognitive flexibility, greater exposure was associated with increased PTSD symptom severity. No such relationship was found in educators with high levels of cognitive flexibility. These results add to a growing body of studies suggesting that flexibility may be a protective factor against the tendency to develop clinical symptoms following stress and traumatic exposure^27,33,73^. For example, a study of fighter pilots revealed that cognitive flexibility extenuated the effect of traumatic exposure on the tendency to develop psychopathological symptoms, including depression, anxiety, and paranoid ideation^73^. Moreover, the current study innovatively shows that this protective role is not limited to general or combat-related traumatic exposure but also exists when referring to stressful incidents that educators may often experience as part of their daily work at school.

When testing the effects of school-related stress exposure and cognitive flexibility on maladaptive coping, we revealed that higher levels of stress exposure were associated with increased maladaptive coping. In contrast, higher levels of cognitive flexibility were associated with decreased levels of maladaptive coping. These findings align with a previous study that showed similar effects in young adults following exposure to a traumatic earthquake^49^. Most importantly, aligning with our hypothesis, we found a significant interactive effect of school-related stress exposure and cognitive flexibility and maladaptive coping. Specifically, for educators with low, but not high, levels of cognitive flexibility, increased school-related stress exposure was associated with higher levels of maladaptive coping. Whereas previous studies highlighted the crucial role of coping abilities for educators^10,40–43^, to the best of our knowledge, this is the first study which demonstrates a more complex picture, which may suggest that cognitive flexibility not only effects clinical symptoms but also determine the way educators behave and function when continuously exposed to school-related stressful events.

The current study provides new insights into the moderating role of cognitive flexibility in the relationship between exposure to school-related stress and PTSD symptoms and coping abilities. Yet, it may suffer possible limitations. First, the majority of the sample is females. This is consistent with the high percentage of women among Israeli educators (above 81%^74^). However, previous studies suggested that women tend to experience more stress than men in similar situations and that their coping style is different. In addition, their tendency to develop PTSD, anxiety, depression, and somatization symptoms is higher than men^74,75^. While important, it should be mentioned that the current study has focused on the relationship between the different variables and that there were no significant differences in the reported measures between women and men in the sample (all *p* > 0.19). With that being said, a future study may aim to test a larger number of male educators.

To summarize, our results highlight the important role of cognitive flexibility when exposed to school-related stress. While its protective role may intuitively be understood, it is important to pay attention to educators with lower levels of cognitive flexibility who may be at a higher risk of developing PTSD symptoms and maladaptive coping. The current study’s findings may pave the way for the development of methods and tools for interventions focused on enhancing educators’ cognitive flexibility. Awareness of its crucial role as a protective factor for educators can serve as a breakthrough in improving educators' well-being and adaptive functioning.

The findings may have important practical implications. Specifically, they may suggest that cognitive flexibility training may help individuals that are exposed to continuous stress as part of their occupational demands, including teachers and other caregivers, to improve coping and decrease their risk for maladaptive outcomes (i.e. decreased PTSD symptoms and maladaptive coping). Such training could be selectively administered to individuals with initially low levels of cognitive flexibility. Previous research indicates that web-based neurocognitive training can enhance cognitive flexibility and reduce symptoms in traumatized individuals with PTSD symptoms^34^. Alternatively, it is possible to use Cognitive Remediation Therapy (CRT), which enables cognitive correction and has been found to be effective in improving cognitive flexibility among adults with anorexia^76^. Finally, studies on Mindfulness-Based Interventions (MBIs) have shown this procedure to be effective in improving cognitive flexibility^77,78^. Since educators are an at-risk population due to their highly stressful work environment^7,8^, such interventions could also prove to be effective in decreasing maladaptive outcomes of prolonged stress exposure in this and similar populations.

## Data Availability

The dataset analyzed during the current study are available in the OSF repository, https://osf.io/sq9mr/?view_only=f2ba6599148d4d57b9a2c55dd7219954.
